# A 9-LncRNA Signature for Predicting Prognosis and Immune Response in Diffuse Large B-Cell Lymphoma

**DOI:** 10.3389/fimmu.2022.813031

**Published:** 2022-07-06

**Authors:** Xiaoxuan Wang, Yaxiao Lu, Ziyi Liu, Yidan Zhang, You He, Cong Sun, Lanfang Li, Qiongli Zhai, Bin Meng, Xiubao Ren, Xudong Wu, Huilai Zhang, Xianhuo Wang

**Affiliations:** ^1^Department of Lymphoma, Tianjin Medical University Cancer Institute and Hospital, National Clinical Research Center for Cancer, Key Laboratory of Cancer Prevention and Therapy, Tianjin’s Clinical Research Center for Cancer, Sino-US Center for Lymphoma and Leukemia Research, Tianjin, China; ^2^State Key Laboratory of Experimental Hematology, The Province and Ministry Co-Sponsored Collaborative Innovation Center for Medical Epigenetics, Key Laboratory of Immune Microenvironment and Disease (Ministry of Education), Department of Cell Biology, School of Basic Medical Sciences, Tianjin Medical University, Tianjin, China; ^3^“5+3” Integration of Clinical Medicine, Tianjin Medical University, Tianjin, China; ^4^Department of Pathology, Tianjin Medical University Cancer Institute and Hospital, Tianjin, China; ^5^Department of Immunology/Biotherapy, Tianjin Medical University Cancer Institute and Hospital, Tianjin, China

**Keywords:** diffuse large B-cell lymphoma, signature, risk score, immune infiltration, prognosis

## Abstract

Diffuse large B-cell lymphoma (DLBCL) is a biologically and clinically heterogeneous disease that requires personalized clinical treatment. To assign patients into different risk categories, cytogenetic abnormalities and genetic mutations have been widely applied to the prognostic stratification of DLBCL. Increasing evidence has demonstrated that deregulated epigenetic modifications and long noncoding RNAs (lncRNAs) contribute to the initiation and progression of DLBCL. However, specific lncRNAs that affect epigenetic regulation and their value in predicting prognosis and therapy response remain uncertain. Here, 2,025 epigenetic-related genes were selected, and 9 lncRNAs (PRKCQ-AS1, C22orf34, HCP5, AC007389.3, APTR, SNHG19, ELFN1-AS1, LINC00487, and LINC00877) were tested and validated to establish an lncRNA-regulating epigenetic event signature (ELncSig). ELncSig, which was established based on independent lymphoma datasets, could distinguish different survival outcomes. Functional characterization of ELncSig showed that it could be an indicator of the immune microenvironment and is correlated with distinctive mutational characteristics. Univariate and multivariate analyses showed that ELncSig was independent of traditional prognostic factors. The novel immune-related ELncSig exhibits promising clinical prognostic value for DLBCL.

## Introduction

Diffuse large B-cell lymphoma (DLBCL) is the most common lymphoid neoplasm in adults. Through cell-of-origin (COO) classification, DLBCL can be identified as activated B-cell-like (ABC), germinal center B-cell-like (GCB), and unclassified subtypes (1). The heterogeneity of DLBCL is reflected in the genetic differences among all subtypes (2). Accumulating evidence indicates that epigenetic regulation plays an important role in DLBCL pathogenesis (3, 4). However, studies of the epigenetic typing of DLBCL are limited. Since epigenetic regulation affects cellular immunity (5), the epigenetic signature of DLBCL is particularly significant.

The tumor microenvironment is important for the growth, invasion, and spread of DLBCL (6–8). The tumor microenvironment is a local pathological environment composed of a variety of cells and biomolecules. Epigenetic regulators play critical roles in DLBCL (9). LncRNAs act in cis or trans to regulate transcription. Recent studies have shown that lncRNAs regulate the interaction between tumor cells and the microenvironment (10), thereby affecting tumor occurrence, development, and metastasis (11). However, research on the role of lncRNAs in lymphoma is not sufficient.

In this study, we developed a novel scoring signature based on lncRNAs to predict the survival outcomes of DLBCL patients. The 9-lncRNA signature provides an improved risk stratification option for patients with DLBCL and sheds new light on potential targeted therapeutic strategies, especially in immunotherapy.

## Materials and Methods

### Patients

#### Collection and Preprocessing of Public Cohort Data

The gene expression data and clinical features of DLBCL samples were collected from the GEO database (http://www.ncbi.nlm.nih.gov/geo/) according to the following selection criteria: (1) basic clinical information on age, gender, IPI score, ECOG-PS, lactate dehydrogenase (LDH) concentration, Ann Anbor stage, extranodal sites, treatment regimen, OS, and survival status; and (2) a large sample size (>300). The GSE10846 (12) and GSE31312 (13) microarray datasets were downloaded.

#### TMUCIH Cohort

The TMUCIH validation cohort enrolled DLBCL patients (*n* = 188) at Tianjin Medical University Cancer Institute and Hospital (TMUCIH; Tianjin, China) from 2008 to 2018. All patients were diagnosed and further confirmed centrally by two experienced pathologists independently (based on the 2008 WHO classification). Patients with complete clinicopathological and follow-up data were included. The major exclusion criteria were as follows: (1) insufficient biopsy material or samples with less than 80% tumor cells, DNA content < 1 μg, and RNA < 5 ng/L; and (2) patients did not have *de novo* DLBCL. The study protocol was approved by the Institutional Review Board of TMUCIH, and all patients provided written informed consent. The reference number of the ethical approval of the current study is bc2021032.

#### Data Integration for the Three Cohorts

The microarray data of 305 (GSE10846) and 404 (GSE31312) samples from DLBCL datasets and TMUCIH (*n* = 160) were used in this study. Samples with high grade B-cell lymphoma with MYC and BCL2 and/or BCL6 rearrangements were excluded. The combat function from the “sva” R package was used to remove the batch effects among different cohorts. A total of 869 patients were eligible, and the clinical information of the patients from the three datasets is shown in **Table 1**.

**Table 1:** Demographic and baseline characteristics of patients enrolled to construct and validate the epigenetic risk score.

	GEO training cohort (GSE10846)	TMUCIH validation cohort	GEO training cohort (GSE31312)
	*n* = 305	*n* = 160	*n* = 404
**Age (years), *n* (%)**			
>60	159 (52.13)	79 (49.38)	238 (58.91)
≤60	146 (47.87)	81 (50.62)	166 (41.09)
**Gender, *n* (%)**			
Male	171 (56.07)	97 (60.62)	235 (58.17)
Female	134 (43.93)	63 (39.38)	169 (41.83)
**ECOG-PS, *n* (%)**			
<2	230 (75.41)	137 (85.63)	342 (84.65)
≥2	75 (24.59)	23 (14.38)	62 (15.35)
**LDH concentration, *n* (%)**			
Normal	157 (51.48)	70 (43.75)	141 (34.90)
Elevated	148 (48.52)	90 (56.25)	263 (65.10)
**Ann Anbor stage, *n* (%)**			
I–II	144 (47.21)	95 (59.38)	193 (47.78)
III–IV	161 (52.79)	65 (40.62)	211 (52.22)
**IPI score, *n* (%)**			
0–2	–	116 (72.5)	258 (63.86)
3–5	–	44 (27.5)	146 (36.14)
**Extranodal sites, *n* (%)**			
<2	140 (45.90)	124 (77.5)	315 (77.97)
≥2	165 (54.10)	36 (22.5)	89 (22.03)
**Treatment, *n* (%)**			
CHOP-like	142 (46.56)	93 (58.13)	–
R-CHOP-like	163 (53.44)	67 (41.87)	404 (100)

Elevated LDH, >245 U/L; ECOG-PS, Eastern Cooperative Oncology Group performance status; LDH, lactate dehydrogenase.

All of the samples from the 160 TMUCIH patients were subjected to targeted deep resequencing using 307 lymphoma-related gene panels (**Supplementary Table S1**) with a total of 26,372 probes and a total probe coverage of 1.666 Mbp. Mutations were identified in 154 of the 160 patients in the TMUCIH cohort (**Supplementary Table S2**).

### Generating the lncRNA-Based Prognostic Signature

For the database samples, we obtained sequencing data from GEO, and we annotated by conversion to the corresponding probe platform ID. Then, a list of epigenetic regulatory genes was generated from GeneCards (https://www.genecards.org /), with a criterion of relevance score >0.5 (*n* = 2,025, **Supplementary Table S3**). To demonstrate that these 2,025 genes have epigenetically related biological functions, functional enrichment was performed by Metascape (14) (https://metascape.org/gp/index.html#/main/step1).

To assess the association between lncRNA expression and OS, we identified lncRNAs regulating epigenetic events (ELncRNAs) by correlation analysis (|r| > 0.4, *p <* 0.01, *n* = 380) (**Supplementary Table S4**). In the GSE10846 cohort, 305 patients were included in a training cohort to generate the prognostic signature. To construct a predictive model, we performed linear regression based on the modified LASSO algorithm using the “glmnet” R package. The ELncSig risk score associated with OS was calculated using the sum of values weighted by the coefficients from the LASSO Cox regression model. The ELncSig score was calculated as follows: (−0.28824 × PRKCQ-AS1 expression) + (0.24206 × C22orf34 expression) − (0.18161 × HCP5 expression) + (0.20887 × AC007389.3 expression) + (0.19686 × APTR expression) + (0.23126 × SNHG19 expression) + (0.33924 × ELFN1-AS1 expression) − (0.13390 × LINC00487 expression) − (0.09065 × LINC00877 expression). Patients were ranked according to the 9-lncRNA signature and dichotomized into high- and low-risk groups.

### Overall Survival Probability Prediction

Receiver operating characteristic (ROC) analysis provides tools to select possibly optimal models and to discard suboptimal ones independently from (and prior to specifying) the cost context or the class distribution. In the case of a balanced diagonal, ROC analysis will tend to the point (0.5, 0.5). Points above the diagonal represent good classification results (better than random); points below the line represent bad results (worse than random). The greater the area under the curve (AUC), the better the survival probability prediction of the model. We also selected clinical characteristics that can be used as independent prognostic factors in multivariate analysis to establish the nomogram (15). The scores corresponding to clinical characteristics can be used to predict patient survival at 1, 3, and 5 years. Model calibration is evaluated by calibration plots of the predicted probability of death at 5 years versus the observed probability. The nomogram-predicted overall survival is plotted on the *x*-axis, with observed overall survival on the *y*-axis. Dashed lines along the diagonal line through the origin point represent perfect calibration models in which the predicted probabilities are identical to the observed probabilities (16).

### Screening for DEGs and Pathway Enrichment

DEGs were identified between the high-risk and low-risk ELncSig groups. The “limma” R package (17) was used in the standard comparison mode. DEG cutoffs were |log_2_FoldChange (log_2_FC)| > 1 and *p <* 0.05. GO functional enrichment and Kyoto Encyclopedia of Genes and Genomes (KEGG) enrichment analyses of the DEGs were performed using the “clusterProfiler” package in R (*p <* 0.05).

### Determination of Immune Cell Infiltration

Immune infiltration was estimated using single-sample gene set enrichment analysis (ssGSEA) (18), and the abundance of 28 immune cell types in the tumor microenvironment was quantified in a range from 0 to 1. The Cell Type Identification by Estimating Relative Subsets of RNA Transcripts (CIBERSORT) algorithm (https://cibersort.stanford.edu/) was used to quantify the relative abundance of 22 immune cell types. After 100 permutations, the gene expression data were quantile normalized.

### Significantly Mutated Genes in Important DLBCL Pathways

The accurate diagnosis of lymphoma relies on gene mutation analysis (19). Considering that each DLBCL patient had different mutation types, we chose 307 lymphoma-related genes and performed targeted gene deep sequencing to determine the genetic compositions of the two ELncSig groups. To determine the differences in important mutated genes, the “maftools” (20) R package was used. The lists of critical pathways and genes in DLBCL were obtained from Young et al. (9).

### Identification of Epigenetic mRNAs Related to ELncSig

A co-expression network of ELncSig including lncRNAs and mRNAs was constructed and visualized using Cytoscape (https://cytoscape.org/). A Sankey diagram showing the associations between the prognostic ELncSig and lncRNAs, mRNAs, and risk type was constructed by the “ggalluvial” R package. The correlations of each ELncRNA with mRNAs are listed in **Supplementary Table S5**.

### Immunohistochemistry Analysis and Evaluation

The use of human remnant DLBCL samples for this study from TMUCIH was approved by the TMUCIH Institutional Review Board (bc2021032). Each biopsy was reviewed by two experienced hematopathologists for diagnostic confirmation. Sections (5 μm thick) of formalin-fixed and paraffin-embedded (FFPE) lymph nodes were dewaxed, hydrated, and heated for antigen retrieval. The cells were blocked with hydrogen peroxide and normal goat serum, incubated overnight with PIM1 (Abcepta, cat# AP7932d, 1:100) and stained with 3,3′-diamino-benzidinetetra hydrochloride (DAB). The PIM1 intensity score was determined as follows: 0—no staining, 1—definite but weak staining, 2—moderate staining, and 3—strong staining. Stained tissue scores were blindly reviewed by two pathologists.

### Statistical Analysis

All statistical and computational analyses were performed with R version 4.0.3 (https://www.r-project.org/). The unpaired Student’s *t*-test was used to compare two clusters with normally distributed variables. Survival outcomes were estimated with the Kaplan–Meier method, and the differences between survival distributions were evaluated by log-rank analysis with the “survival” package in R software. The Wilcoxon test was used to compare two clusters with nonnormally distributed variables. Contingency table variable analysis was completed by two-sided Fisher’s exact tests. We used the ELncSig risk score and clinical characteristic covariates to construct a nomogram to estimate survival. The accuracy of the nomogram was measured using the calibration curve. Univariate and multivariate analyses of prognosis were evaluated using a Cox proportional hazards regression model. The statistical significance cutoff was set at *p <* 0.05.

## Results

### Construction of an Epigenetic-Related lncRNA Risk Signature

**Figure 1:**
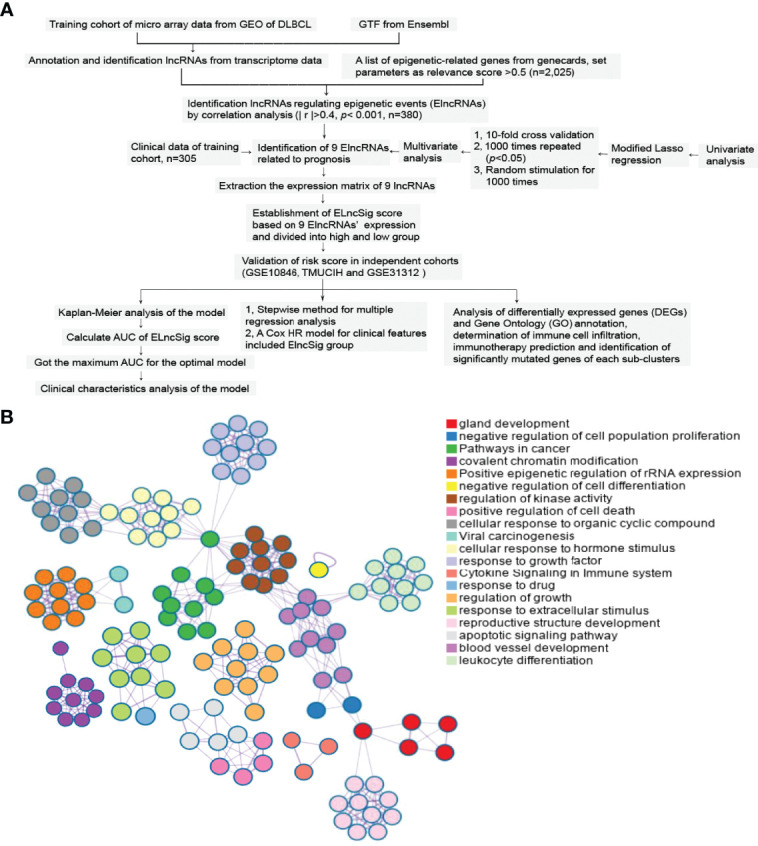
Study flowchart and epigenetic-related gene enrichment pathways. **(A)** The workflow of the study. **(B)** Different colors indicate the 2,025 epigenetic-related gene annotations and biological processes provided by Metascape.

After removing the clinical samples meeting the exclusion criteria, a total of 869 samples were included as the subjects of this study. The study flowchart is shown in **Figure 1A**. Metascape analyses showed the diverse biological processes of 2,025 genes (**Figure 1B**).

**Figure 2:**
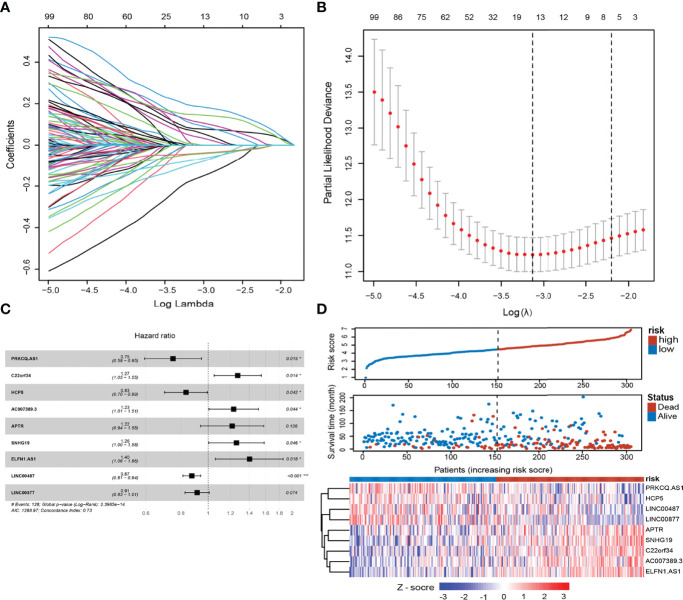
Construction of the epigenetic-related lncRNA signature (ELncSig). **(A)** Thirteen epigenetic-related lncRNAs were selected by LASSO Cox regression analysis. **(B)** Cross-validation for tuning parameter selection in the proportional hazards model. **(C)** A forest map showing 9 lncRNAs identified by the stepwise method. **(D)** Risk score distribution, survival status, and lncRNA expression of DLBCL patients in high- and low-risk groups classified by the 9-ELncRNA signature in the training cohort.

Through Pearson correlation analysis, we identified 380 ELncRNAs (|*r*| > 0.4, *p <* 0.01), which were then subjected to univariate analysis. After modified LASSO regression analysis with tenfold cross-validation, repeated 1,000 times (*p <* 0.05) with random simulation 13 ELncRNAs were extracted (**Figures 2A, B**). After multivariate analysis, 9 lncRNAs were tested and validated to establish ELncSig (**Figure 2C**, **Supplementary Table S6**). A heatmap of the expression levels of the 9 identified lncRNAs and a scatterplot of OS with relevant risk scores are presented in **Figure 2D**. Among the 9 lncRNAs, 5 lncRNAs were identified as poor prognostic factors (**Supplementary Figure S1A**), while another 4 were identified as favorable prognostic factors (**Supplementary Figure S1B**).

### Evaluation of ELncSig as an Independent Prognostic Factor for DLBCL

**Figure 3:**
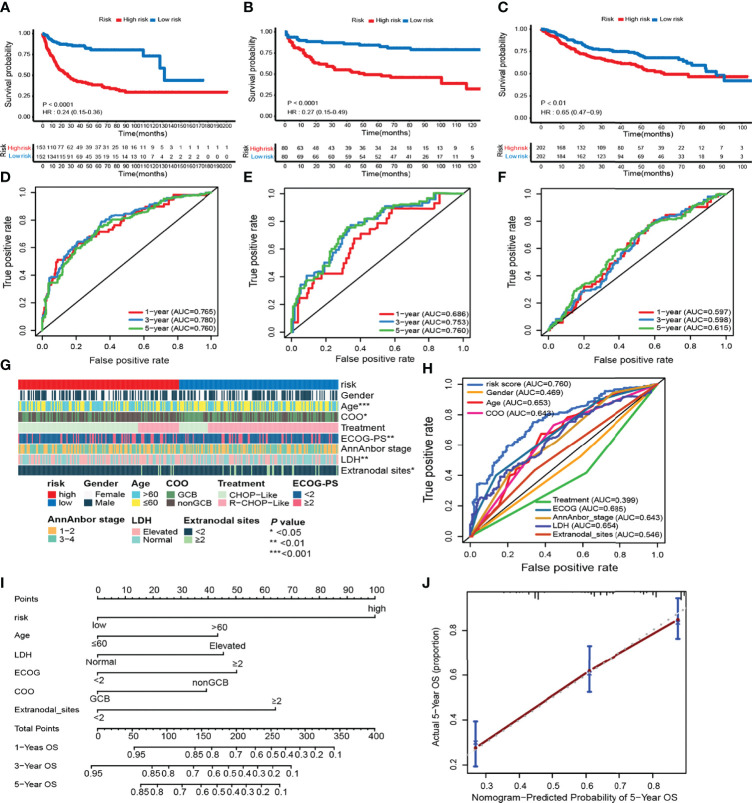
Prognostic value of the risk model including 9 epigenetic-related lncRNAs. **(A–F)** Kaplan–Meier survival curves of the high- and low-risk groups and time-dependent receiver operating characteristic (ROC) curves at 1-, 3-, and 5-year overall survival (OS) in the training (GSE10846) and validation (TMUCIH and GSE31312) cohorts. **(G)** Heatmap showing the comparison of the clinicopathological characteristics of DLBCL patients in the high- and low-risk GSE10846 groups. **(H)** Time-dependent ROC curve analyses for predicting OS at 5 years with clinicopathological characteristics. **(I)** The nomogram was constructed using high and low ELncSig scores, age, LDH, GCB vs. non-GCB, ECOG, and extranodal sites to predict 1-, 3-, and 5-year survival. **(J)** Calibration plots for the probability of 5-year survival in the training cohort.

To identify the efficacy of ELncSig for DLBCL survival prediction, the training cohort samples were divided into a low-risk group (*n* = 152) and a high-risk group (*n* = 153) using the median risk score as a cutoff point. Low-risk patients had significantly better OS than high-risk patients (**Figure 3A**). Kaplan–Meier analysis in the internal validation cohort also indicated that ELncSig could be a good prognostic factor (**Figure 3B**). This association remained markedly significant in the multivariate Cox model in the training and validation cohorts (**Table 2**). Data from another external validation cohort from GSE98588 are shown in **Supplementary Figure S2**. One external validation cohort (GSE31312) only contained patients treated with an R-CHOP-like regimen, and their survival could be acceptably stratified by the ELncSig risk score (**Figure 3C**). Because the risk score model was developed based on all patients, to verify its reliability, we tested the model in patients who were only treated with an R-CHOP-like regimen in the training cohort (GSE10846) and the external validation cohort (GSE98588). The ELncSig model was effective in these patient populations treated with R-CHOP (**Supplementary Figure S3**).

**Table 2:** Univariate and multivariate Cox regression analysis of predictors of survival outcomes in the training and validation cohorts.

	Univariate Analysis		Multivariate Analysis	
Variable	*p*	HR (95% CI)	*p*	HR (95% CI)
**GSE10846 training cohort**				
**Age (>60 vs. ≤60)**	<0.001	0.48 (0.330–0.697)	0.003	0.56 (0.38–0.82)
**Gender (Female vs. Male)**	0.659	0.92 (0.646–1.318)	0.908	1.02 (0.71–1.47)
**COO class (GCB vs. nonGCB)**	<0.001	2.57 (1.724–3.839)	0.018	1.65 (1.09–2.50)
**LDH concentration** **(Elevated vs. Normal)**	<0.001	0.41 (0.280–0.591)	0.007	0.57 (0.38–0.86)
**ECOG-PS (≤1 vs. ≥2)**	<0.001	2.77 (1.916–4.004)	0.002	1.86 (1.24–2.78)
**Rituximab (No vs. Yes)**	0.001	0.54 (0.366–0.787)	0.956	1.01 (0.62–1.65)
**Extranodal sites (<2 vs. ≥2)**	0.053	1.86 (0.993–3.471)	0.038	2.13 (1.04–4.35)
**ELncSig (high risk vs. low risk)**	<0.001	0.24 (0.155–0.364)	<0.001	0.26 (0.16–0.43)
**TMUCIH validation cohort**				
**Age (>60 vs. ≤60)**	0.028	0.56 (0.329–0.937)	0.036	0.53 (0.29–0.96)
**Gender (Female vs. Male)**	0.445	1.23 (0.722–2.099)	0.748	0.91 (0.51–1.62
**ECOG-PS (≤1 vs. ≥2)**	0.407	1.33 (0.675–2.634)	0.267	1.53 (0.72–3.26)
**LDH concentration** **(Elevated vs. Normal)**	0.002	0.43 (0.257–0.726)	0.274	0.68 (0.34–1.36)
**COO class (GCB vs. nonGCB)**	0.073	1.61 (0.956–2.727)	0.002	2.44 (1.21–4.21)
**IPI score (0–2 vs. 3–5)**	<0.001	2.91 (1.741–4.868)	0.795	0.88 (0.35–2.25)
**Rituximab (No vs. Yes)**	0.176	0.69 (0.402–1.181)	0.093	0.62 (0.35–1.08)
**Ann Anbor stage** **(I–II vs. III–IV)**	0.012	1.93 (1.156–3.215)	0.540	1.24 (0.62–2.49)
**Extranodal sites (<2 vs. ≥2)**	<0.001	2.88 (1.691–4.912)	0.011	2.26 (1.21–4.21)
**ELncSig (high risk vs. low risk)**	<0.001	0.28 (0.153–0.495)	<0.001	0.28 (0.14–0.54)

HR, hazard ratio; CI, confidence interval; ECOG-PS, Eastern Cooperative Oncology Group performance status; RCHOP, rituximab plus cyclophosphamide, vincristine, doxorubicin, and prednisone; LDH, lactate dehydrogenase; COO, cell of origin; GCB, germinal center B-cell like.

Next, we calculated the AUCs for each ROC curve to assess the predictive accuracy of the model. The AUC value is often used as the evaluation criterion for a model (21). In time-dependent ROC analysis at 1, 3, and 5 years, the AUC values were 0.765, 0.780, and 0.760, respectively (**Figure 3D**). For the validation cohorts, higher AUC values were obtained for 5-year survival (**Figures 3E, F**).

The tumor-related clinicopathological features of the two ELncSig groups were evaluated in the training cohort. We found that the patient age (*p =* 0.003), plasma lactate dehydrogenase (LDH) levels (*p =*0.007), Eastern Cooperative Oncology Group performance status (ECOG-PS, *p =* 0.002), COO classification (*p =* 0.018), and extranodal sites (*p =* 0.038) were significantly correlated with ELncSig (**Figure 3G**, **Supplementary Table S7**). The tumor-related clinicopathological features in these datasets were inferior to ELncSig (**Figure 3H**) in the training cohort. A nomogram for 1-, 3-, and 5-year mortality was constructed (**Figure 3I**), and the calibration for 5 years indicated that the mortality estimated by the nomogram was close to the actual mortality (**Figure 3J**). Hence, ELncSig predicts 5-year survival better. The validation cohort results are shown in **Supplementary Figure S4**.

### Identification of DEGs Between the ELncSig Groups

**Figure 4:**
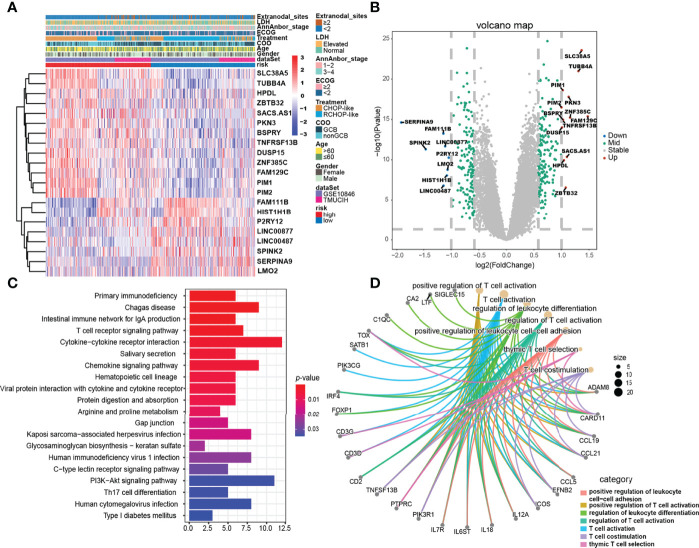
Gene expression differences and relevant biological pathways between the high- and low-risk ELncSig groups. **(A)** Heatmap of differentially expressed genes between the high- and low-risk ELncSig groups and their clinicopathological characteristics. **(B)** The volcano plot showing upregulated and downregulated genes between the two groups (|log_2_FC| > 1, adjusted *p* < 0.05). **(C)** Heatmap showing the KEGG pathways enriched in the high- and low-risk ELncSig groups. **(D)** Circle map showing the immune-related pathways regulated by DEGs between the two groups and the genes included in the pathways.

To uncover the biological distinction between the two ELncSig groups, we performed DEG analysis in the GSE10846 and TMUCIH cohorts combined. The heatmap of the DEGs between the high-risk and low-risk ELncSig groups is shown in **Figure 4A** (|fold change| >1.5, adjusted *p <* 0.01). After comparing the high-risk group with the low-risk group, 172 upregulated and 154 downregulated genes were identified (**Supplementary Table S8**). We found that the primary central nervous system DLBCL-related protein HPDL, the tumor necrosis factor receptor superfamily member TNFRSF13B, and the serine/threonine protein kinase family members PIM1 and PIM2 were upregulated in the high-risk group, while the histone gene HIST1H1B and protective lncRNAs in ELncSig, such as LINC00487 and LINC00877, were downregulated in the high-risk group (**Figure 4B**).

Subsequently, KEGG enrichment analysis of the DEGs indicated enrichment of primary immunodeficiency, the T-cell receptor signaling pathway, cytokine–cytokine receptor interaction, the PI3K-Akt signaling pathway, and Th17-cell differentiation (**Figure 4C**, **Supplementary Table S9**). GO enrichment analysis indicated enrichment of genes involved in positive regulation of leukocyte cell–cell adhesion, regulation of T-cell activation, and T-cell co-stimulation (**Figure 4D**, **Supplementary Table S10**). After GSEA of the high-risk and low-risk groups, similar results were found, as shown in **Supplementary Figure S5**.

### Estimation of the Tumor-Infiltrating Immune Cells of the Two ELncSig Groups

**Figure 5:**
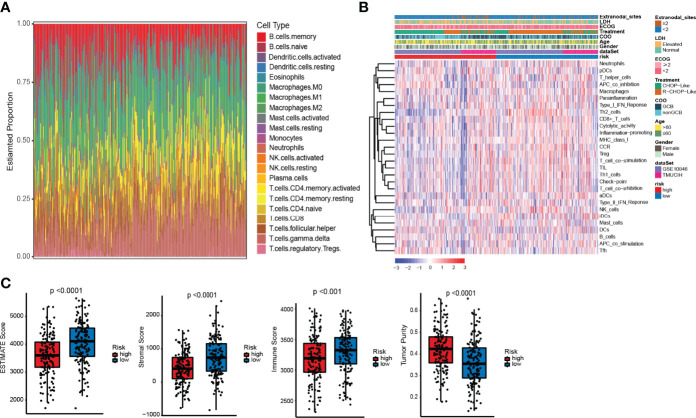
Differences in immune infiltration between the high- and low-risk ELncSig groups. **(A)** The composition of immune cells assessed by the Cell Type Identification by Estimating Relative Subsets of RNA Transcripts (CIBERSORT) algorithm in the training cohort. **(B)** Heatmap showing the relative abundances of 28 infiltrating immune cell subpopulations between the high- and low-risk ELncSig groups according to single-sample gene set enrichment analysis (ssGSEA). **(C)** The ESTIMATE score, stromal score, immune score, and tumor purity of EC1 and EC2 according to the CIBERSORT algorithm.

The pathway enrichment results indicated that the different prognoses of ELncSig are closely associated with immune infiltration. We assessed the composition of tumor-infiltrating immune cells in DLBCL samples by the CIBERSORT algorithm (22). The histogram showed that memory B cells, M0 macrophages, and T cells were obviously highly abundant in DLBCL samples (**Figure 5A**), and they might play essential roles in the initiation and development of DLBCL (23, 24). Using ssGSEA to show the different immune components between the two ELncSig groups, we found that compared with the high-risk group, CD8^+^ T cells, T helper cells, macrophages, Th1 and Th2 cells, CCR, and T-cell co-stimulatory cells were enriched in the low-risk ELncSig group (*p <* 0.05, **Figure 5B**). We further compared the ESTIMATE score, stromal score, immune score, and tumor purity (25). As shown in **Figure 5C**, the low-risk ELncSig group had significantly higher ESTIMATE scores, stromal scores, and immune scores and a lower tumor purity (*p <* 0.001).

### Potential of ELncSig as an Indicator of Immunotherapy Response in DLBCL Patients

**Figure 6:**
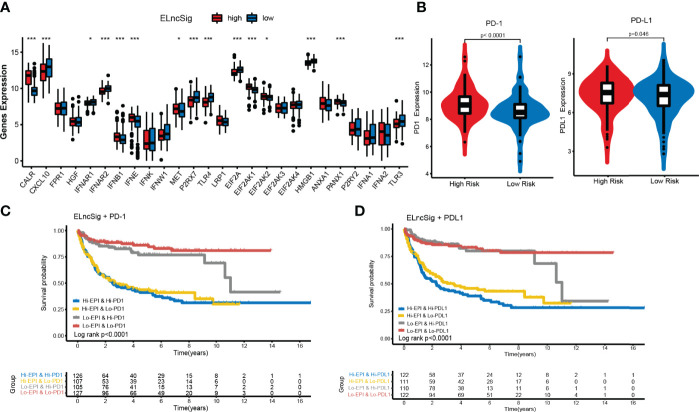
Impact of immunogenic cell death (ICD) modulators and immune checkpoint gene expression on clinical outcome. **(A)** Differential expression of ICD modulators between the high- and low-risk ELncSig groups. **(B)** Immune checkpoint expression of PD-1 and PD-L1. **(C, D)** Kaplan–Meier survival curves of overall survival among the four patient groups stratified by ELncSig and PD-1 and PD-L1. *p < 0.05, **p < 0.01, ***p < 0.001.

Immunogenic cell death (ICD) and immune checkpoints (ICPs) play important roles in the tumor immune microenvironment (26–28). As shown in **Figure 6A**, the expression levels of various ICD genes, such as CXCL10, IFNAR2, P2RX7, TLR4, EIF2A, HMGB1, and TLR3, were significantly upregulated in the low-risk ELncSig group. Similar to ICD genes, ICPs can also reflect the immune status of the tumor microenvironment. The PD-1 and PD-L1 checkpoints were highly expressed in the high-risk ELncSig group (**Figure 6B**). The survival distribution of the two patient groups stratified by ELncSig and high/low ICP gene expression was compared. As shown in **Figures 6C, D**, patients with low ELncSig and high PD-1/PD-L1 had significantly better survival than those with high ELncSig and high PD-1/PD-L1 (log-rank *p <* 0.0001), and patients with low ELncSig and low PD-1 also had prolonged survival relative to those with high ELncSig and low PD-1 (log-rank *p <* 0.0001). Similar results for two other important checkpoints, TNFRSF4 and IDO1, are shown in **Supplementary Figure S6**.

### Differences In Important Gene Mutations Between the Two ELncSig Groups

**Figure 7:**
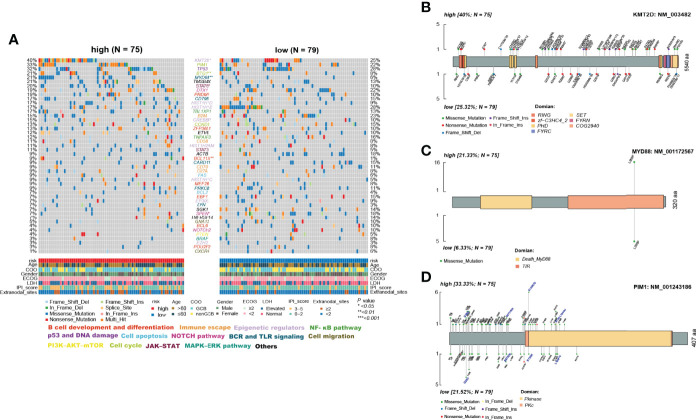
Differences in somatic mutations between the high- and low-risk ELncSig groups in the TMUCIH validation cohort. **(A)** Oncoplot analysis of critical mutated genes and pathways in DLBCL between the high- and low-risk ELncSig groups (two-sided Fisher’s exact test). **(B–D)** Specific mutated site analysis of KMT2D, MYD88, and PIM1.

The use of cytogenetic abnormalities and genetic mutations for the prognostic stratification of DLBCL and assignment of patients into different risk categories has been widely studied (2, 29). We investigated whether critical differences in pathways related to somatic mutation frequencies exist between the two groups. Because the online databases lack sufficient mutation information, we further analyzed significantly mutated genes in the TMUCIH validation cohort by performing targeted deep resequencing of 307 lymphoma-related gene panels. Based on the results of previous studies, we established a mutational landscape of the important genes in DLBCL (**Figure 7A**, **Supplementary Table S11**).

Abundant genetic alterations in various critical pathways, such as the epigenetic regulator pathway (KMT2D, *p =* 0.034), the BCR and TLR signaling pathway (MYD88, *p =* 0.003), B-cell development and differentiation (BCL11A, *p =* 0.02308), and the cell cycle pathway (BTG1, *p =* 0.009), were significantly enriched in the high-risk ELncSig group. Interestingly, SPEN (*p =* 0.027) in the NOTCH pathway was mutated more frequently in the low-risk ELncSig group.

KMT2D (lysine methyltransferase 2D, MLL2), a chromatin epigenetic modifier, plays a vital role in modulating ICP blockade (30). MYD88 mutation is one of the most remarkable drivers in the development of DLBCL (31), and the L265P mutation is now thought to be common to virtually all NHLs and occurs in between 4% and 90% of cases, depending on the entity (32). PIM1, as a DEG and a high-frequency gene in DLBCL, also affects the prognosis of patients (33) and has a trend of mutational differences between the two groups. Hence, we selected these three genes to analyze their specific mutation sites. It was obvious that in the high-risk group, PIM1 had more vital mutation sites in S97T, E135Q, and K183-L184del, and it has already been reported that mutated PIM1 may lead to a poor prognosis (34) (**Figures 7B–D**).

### Verification of ELncSig-Influenced mRNAs

**Figure 8:**
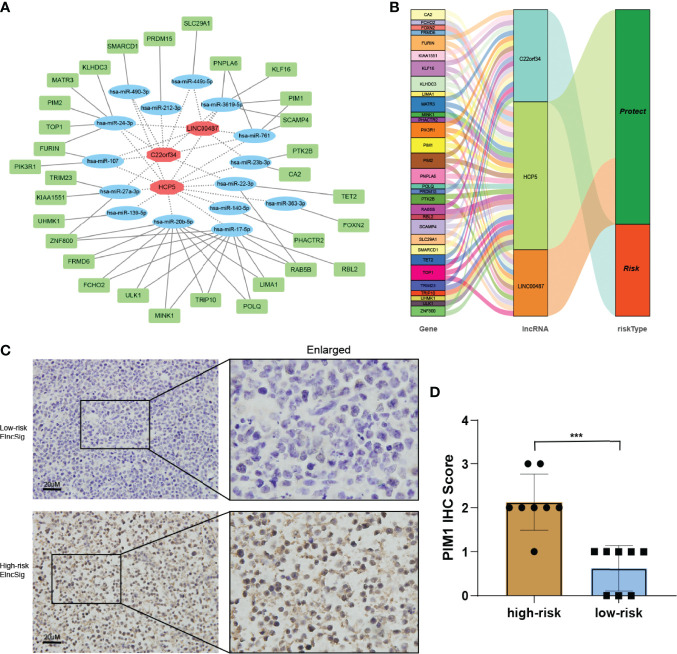
Co-expression network and validation of prognostic ELncSig lncRNAs and the associated genes. **(A)** A co-expression network of ELncSig lncRNAs and mRNAs was constructed and visualized using Cytoscape. The red hexagons indicate prognostic lncRNAs, and the green rectangles indicate ELncSig mRNAs. **(B)** Sankey diagram showing the associations between prognostic ELncSig lncRNAs, mRNAs, and risk type. **(C, D)** Immunohistochemical images and differential analysis of PIM1 in high- and low-risk ELncSig (***p < 0.001, by Student's t-test).

We built a ceRNA network on the basis of the expression profiles of miRNAs and ELncSig-included lncRNAs and mRNAs in patients with DLBCL. In total, 3 lncRNA nodes, 15 miRNA nodes, and 31 mRNA nodes were identified as differentially expressed profiles (|fold change| >1.5, *p <* 0.05, **Figure 8A**). In the present study, a ceRNA network containing 2,025 genes affecting epigenetic regulation was constructed (**Supplementary Figure S7A**). Once again, the 2,025 epigenetic regulatory genes affected by these lncRNAs and their corresponding risk groups were identified. We observed that TET2, E2F1, KDM1A, HDAC7, and KMT2A were regulated by ELncSig lncRNAs (**Figure 8B**, **Supplementary Figure S7B**). PIM1, PIM2, and PIK3R1 were also affected, which means that ELncSig could not only regulate epigenetic-related genes but also affect genes related to other pathways.

PIM1 is a gene regulated by ELncSig and has significantly different mRNA levels between the high- and low-risk groups. Strikingly, high PIM1 expression was significantly correlated with the high-risk ELncSig group (**Figures 8C, D**).

## Discussion

Most studies have focused on establishing a new signature of protein-coding genes in DLBCL (35). Based on the 7 subtypes constructed by Wright et al., a probabilistic classification tool for DLBCL genotypes (LymphGen algorithm) was proposed, and 63.1% of tumors can be identified by their genotypes (29). Establishing these signatures mostly relies on quantifying gene transcript levels. We were inspired to show that epigenetic genes play an important role in lymphoma and affect immunity through immune-related gene pairing and attempted to construct a reasonable prognostic model using 9 lncRNAs that are closely correlated with epigenetic-related gene combinations (10, 11). We did not use their expression values at the beginning of signature construction.

In general, high-abundance lncRNAs possess significant biological functions (36). Our findings suggest that ELncSig can be used to identify epigenetic-related genes and predict patient prognosis. In addition, lncRNAs can efficiently pair with protein-coding genes. Our model can distinguish between high- and low-clinical risk patients with the advantage of clinical practicability. Because lncRNAs are associated with immune infiltration, it is reasonable for them to affect the immune microenvironment and the activation of immune cells and to be predictable of the response to immune therapy. In fact, studies have already found that PRKCQ-AS1 and HCP5, which are included in our ELncSig, play important roles in the process of lymphoma (37, 38), while other lncRNAs were revealed in DLBCL for the first time. Blandino et al. reported that C22orf34 expression gradually decreased from gallstones to gallbladder cancer (39). Guan et al. showed that APTR contributes to osteosarcoma progression through repression of miR-132-3p and upregulation of YAP1 (40), and Zhou et al. reported that APTR promotes uterine leiomyoma cell proliferation by targeting ERa to activate the Wnt/β-Catenin pathway (41). SNHG19 and ELFN1-AS1 have been used to predict the survival of triple-negative breast cancer and non-small cell lung cancer, respectively (42, 43). LINC00487 was shown to be a protective factor in hepatocellular carcinoma (44), and LINC00877 was found to have lower expression in bone marrow samples (45). Some of the roles of these 9 ELncRNAs in solid tumors are similar to those in DLBCL, while others are different. In summary, these lncRNAs are associated with the occurrence and development of tumors; hence, the proposed model can identify novel biomarkers for further research in DLBCL.

We referred to the modified LASSO model used by Sveen et al. (46) to construct the initial signature system. In the process of inclusion in the Cox regression model, the factors were ranked according to their frequency, which suggests the impact of the factor on the model. We assessed the ELncSig risk model using a QQ test and found a normal distribution. Thus, we used the median value to separate patients into high- and low-risk groups. Subsequently, we performed univariate and multivariate analyses of clinicopathological characteristics, calculated AUC values, constructed a nomogram, and assessed its calibration to evaluate the robustness of this model. After analyzing survival outcomes, clinical features, tumor immune infiltration, biomarkers related to checkpoint inhibitors, immune therapy predictions, mutations, the constructed ceRNA network, and immunohistochemical confirmation, the results implied that this ELncSig model worked well in the training and validation cohorts.

Mutations in the gene encoding the KMT2D (or MLL2) methyltransferase are highly recurrent and occur early during tumorigenesis in DLBCL. DLBCL-associated KMT2D mutations impair KMT2D enzymatic activity, leading to diminished global H3K4 methylation in GCB cells and DLBCL cells (47), and KMT2D could be a modulator of ICP blockade (30). For MYD88, L265P is a gain-of-function driver mutation. The L265P mutant promotes cell survival by spontaneously assembling a protein complex containing IRAK1 and IRAK4 (48). PIM1 belongs to the PIM kinase family and has been proven to exhibit ABC-associated mutations (49). We also explored the specific mutation sites of these important molecules in DLBCL and further clarified the reason why the high-risk group had a poor prognosis.

In recent years, immunotherapies based on checkpoint inhibitors have shown promising results in the treatment of aggressive malignancies, including Hodgkin’s lymphoma (27). PD-L1 overexpression has also been observed in the aggressive ABC/non-GCB subtype of DLBCL (50). To explore the relationship between ELncSig and tumor-infiltrating immune cells, we used three common methods to estimate immune-infiltrating cells: ESTIMATE, CIBERSORT, and ssGSEA. We found that the low-risk ELncSig group was more positively related to tumor-infiltrating immune cells, such as CD8^+^ T cells, macrophages, Th2 cells, and major histocompatibility complex (MHC) class I. Subsequent immune-related scores also showed that the low-risk ELncSig group had a better immune microenvironment. When tumor ICD is induced, the ratio of cytotoxic T lymphocytes (CTLs) to Tregs in the tumor increases, indicating good patient prognosis. In contrast, a decrease in this ratio may suggest a poor prognosis (51). Similar to ICDs, ICPs can also reflect the immune status of tumor microenvironments. In the present study, significantly prolonged survival was observed for patients with low ELncSig and low ICP gene expression, implying that these patients with low ELncSig may have a better response to ICP therapy.

## Conclusion

We constructed an lncRNA signature based on epigenetic-related genes to predict the prognosis of DLBCL. We also proved that this new signature could affect other coding proteins in addition to epigenetic genes. Importantly, ELncSig might be associated with immune infiltration levels and even the efficacy of tumor immunotherapy.

## Data Availability Statement

Training and external validation samples were obtained from the GEO online datasets GSE10846, GSE31312 and GSE98588. All data generated or analyzed during this study are included in this published article and its supplementary information files. Additional related-data are available from the corresponding author upon reasonable request.

## Ethics Statement

The studies involving human participants were reviewed and approved by Tianjin Medical University Cancer Institute and Hospital. The patients/participants provided their written informed consent to participate in this study.

## Author Contributions

XXW, XDW, XHW, and HLZ contributed to the study conception and design. XXW, YDZ, YXL, and ZYL collected and analyzed the data. LFL, YH, and CS performed data acquisition. QLZ and BM reviewed the pathology results. XBR provided technical help. XXW and XHW drafted the manuscript. XDW, XHW, and HLZ reviewed and revised the manuscript. XHW and HLZ contributed to study supervision. All authors read and approved the final manuscript.

## Funding

This study was supported by grants from the Natural Science Foundation of Tianjin (19JCYBJC26500), the National Natural Science Foundation of China (81770213), and the Clinical Oncology Research Fund of CSCO (Y-XD2019-162).

## Conflict of Interest

The authors declare that the research was conducted in the absence of any commercial or financial relationships that could be construed as a potential conflict of interest.

## Publisher’s Note

All claims expressed in this article are solely those of the authors and do not necessarily represent those of their affiliated organizations, or those of the publisher, the editors and the reviewers. Any product that may be evaluated in this article, or claim that may be made by its manufacturer, is not guaranteed or endorsed by the publisher.

## Supplementary Material

Supplementary Figure 1 Kaplan–Meier analysis of 9 prognosis-related lncRNAs.

Supplementary Figure 2 Validation of ELncSig in the GSE98588 cohort. **(A)** A forest plot showing the 9 lncRNAs identified by the stepwise method in the external validation cohort. **(B)** Risk score distribution, survival status and lncRNA expression of DLBCL patients in the high- and low-risk groups classified by the 9-ELncRNA signature in the external validation cohort. **(C)** Kaplan-Meier analysis of the different risk groups. **(D)** Time-dependent receiver operating characteristic (ROC) curves for 1-, 3-, and 5-year overall survival (OS) in the external validation cohorts.

Supplementary Figure 3 Validation of ELncSig performance in two external cohorts treated with R-CHOP-like regimens. **(A)** ELncSig performance for samples from patients treated with R-CHOP-like regimens in the GSE10846 dataset. **(B)** ELncSig performance for samples from patients treated with R-CHOP-like regimens in the GSE98588 dataset.

Supplementary Figure 4 Validation of ELncSig in the TMUCIH cohort. **(A)** Risk score distribution, survival status and lncRNA expression of DLBCL patients in the high- and low-risk groups classified by the 9-ELncRNA signature in the TMUCIH validation cohort. **(B)** A nomogram was constructed using high and low ELncSig scores, age, LDH, GCB vs. non-GCB, ECOG and extranodal sites to predict 1-, 3- and 5-year survival. **(C)** Calibration plots for the probability of five-year survival in the training cohort. **(D)** Time-dependent ROC curve analyses for predicting OS at 5 years with clinicopathological characteristics.

Supplementary Figure 5 GSEA of the DEGs in the training cohort.

Supplementary Figure 6 Immune checkpoint genes related to clinical outcome. **(A, B)** Kaplan–Meier survival curves of overall survival among four patient groups stratified by ELncSig and TNFRSF4 and IDO1.

Supplementary Figure 7 Coexpression network and validation of prognostic ELncSig lncRNAs and the associated epigenetic-related genes. **(A)** A coexpression network of ELncSig lncRNAs and mRNAs (only epigenetic-related genes) was constructed and visualized using Cytoscape. Red hexagons indicate prognostic lncRNAs, and green rectangles indicate ELncSig mRNAs. **(B)** Sankey diagram showing the associations among prognostic ELncSig lncRNAs, mRNAs (only epigenetic-related genes), and risk type.

Supplementary Table 1 The 307 gene panel design.

Supplementary Table 2 Targeted gene deep sequencing of the TMUCIH validation cohort.

Supplementary Table 3 Epigenetic related genes from GeneCards with relevance scores >0.5.

Supplementary Table 4 The 380 epigenetic related lncRNAs list.

Supplementary Table 5 The correlation between epigenetic genes and lncRNAs.

Supplementary Table 6 The 9 epigenetic-related genes used for multivariate Cox regression in the training and validation cohorts.

Supplementary Table 7 Multivariate Cox regression of tumor-related clinicopathological features.

Supplementary Table 8 Differentially expressed gene results.

Supplementary Table 9 KEGG enrichment of DEGs for two merged cohorts.

Supplementary Table 10 GO enrichment of DEGs for two merged cohorts.

Supplementary Table 11 Gene alterations in the TMUCIH validation cohort.
