# Supplementary figures and images for: A 9-LncRNA Signature for Predicting Prognosis and Immune Response in Diffuse Large B-Cell Lymphoma
Source: Front Immunol. 2022 Jul 6;13:813031. doi: 10.3389/fimmu.2022.813031 (PMC9298982; doi:10.3389/fimmu.2022.813031)

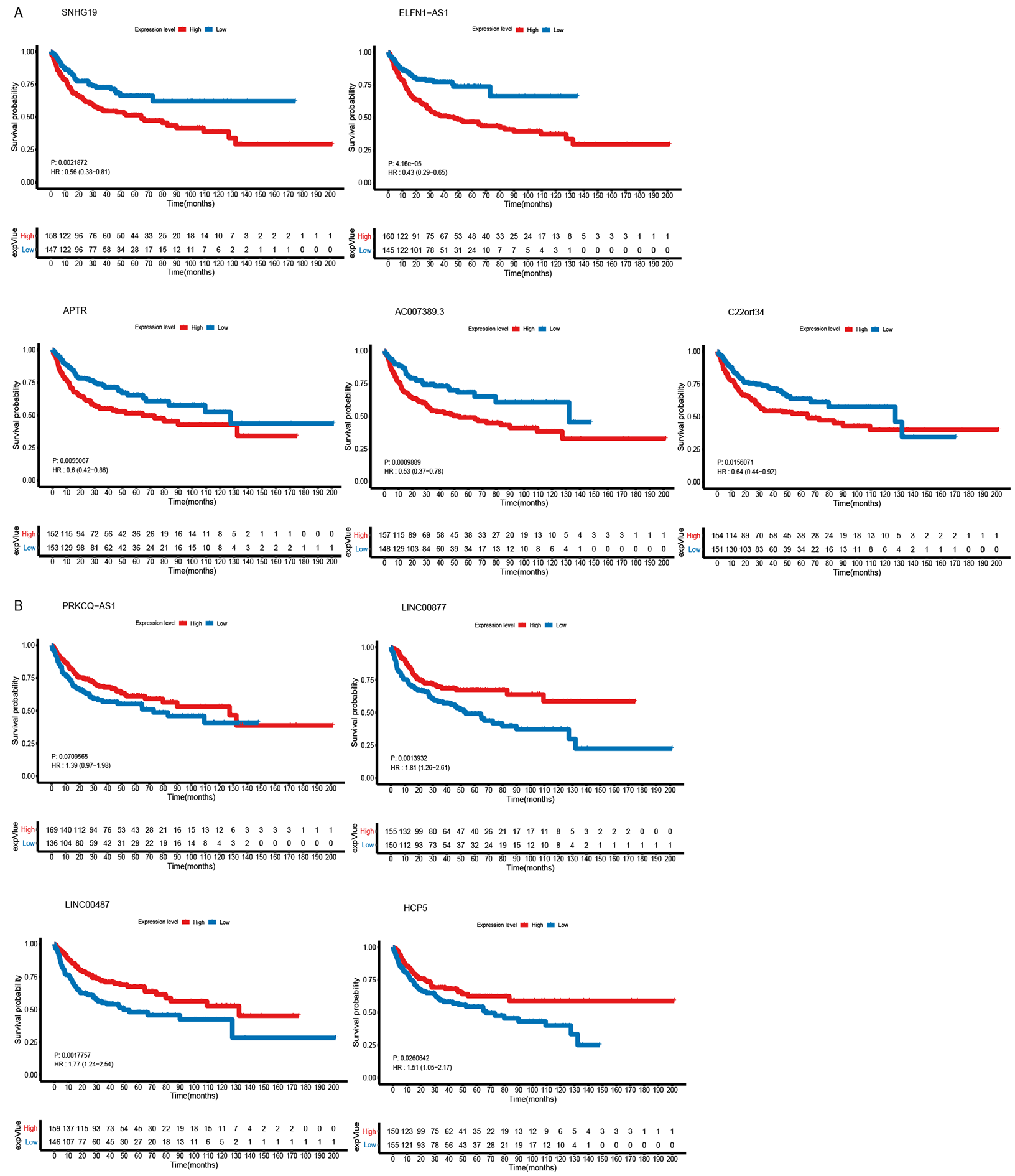

Supplement: Supplementary Figure 1 — Kaplan–Meier analysis of 9 prognosis-related lncRNAs. [file Image_1.tif]

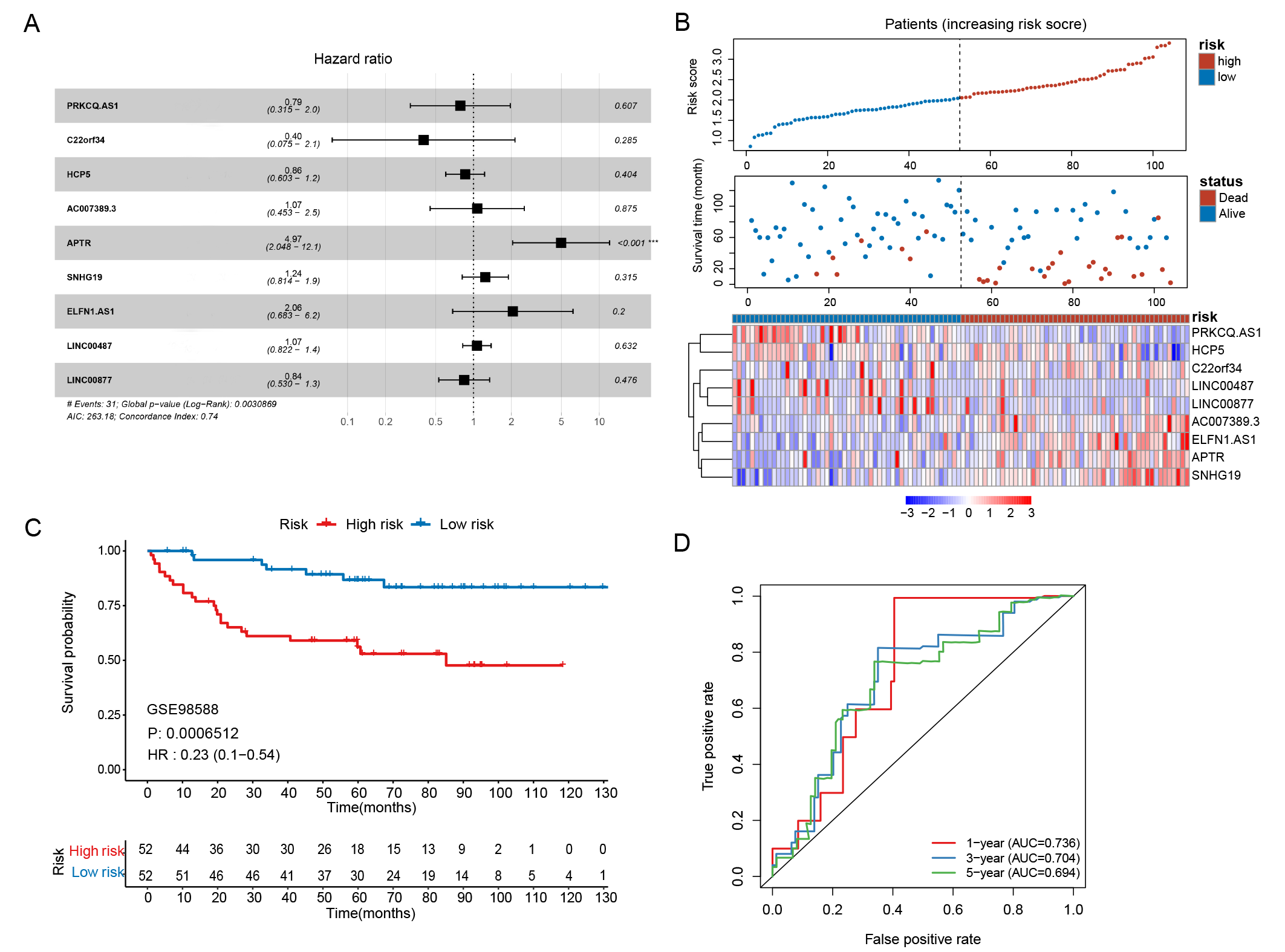

Supplement: Supplementary Figure 2 — Validation of ELncSig in the GSE98588 cohort. (A) A forest plot showing the 9 lncRNAs identified by the stepwise method in the external validation cohort. (B) Risk score distribution, survival status and lncRNA expression of DLBCL patients in the high- and low-risk groups classified by the 9-ELncRNA signature in the external validation cohort. (C) Kaplan-Meier analysis of the different risk groups. (D) Time-dependent receiver operating characteristic (ROC) curves for 1-, 3-, and 5-year overall survival (OS) in the external validation cohorts. [file Image_2.tif]

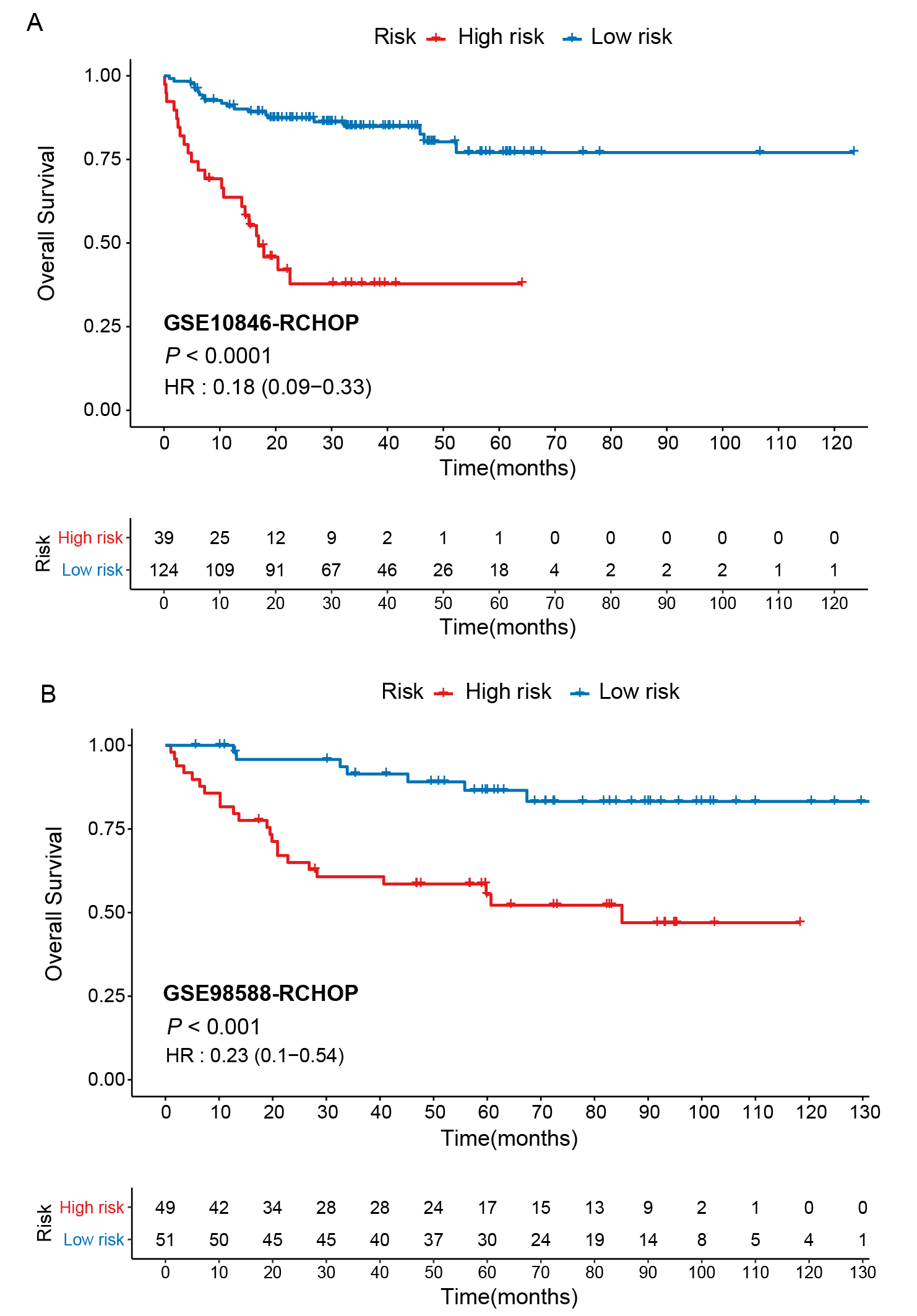

Supplement: Supplementary Figure 3 — Validation of ELncSig performance in two external cohorts treated with R-CHOP-like regimens. (A) ELncSig performance for samples from patients treated with R-CHOP-like regimens in the GSE10846 dataset. (B) ELncSig performance for samples from patients treated with R-CHOP-like regimens in the GSE98588 dataset. [file Image_3.tif]

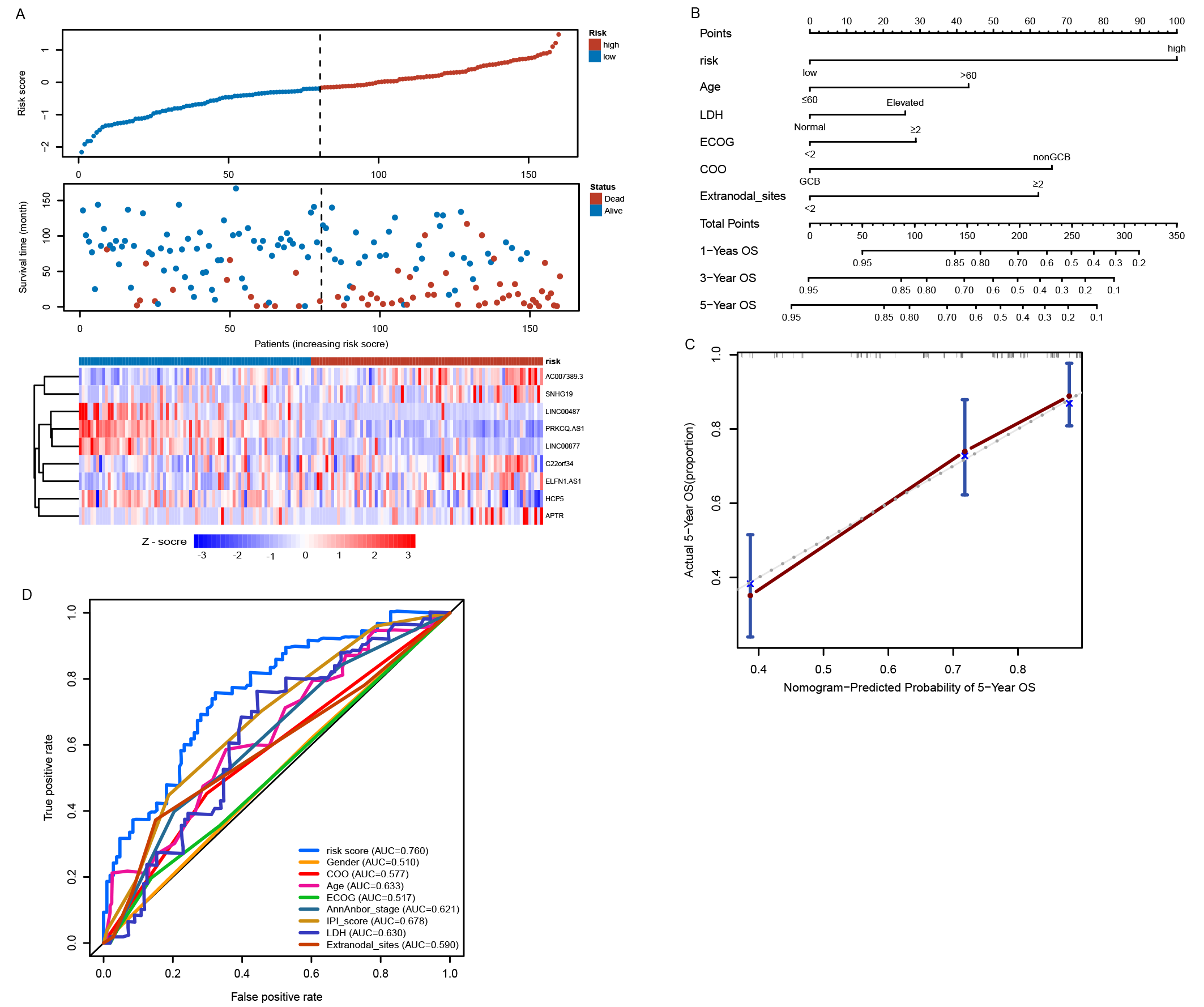

Supplement: Supplementary Figure 4 — Validation of ELncSig in the TMUCIH cohort. (A) Risk score distribution, survival status and lncRNA expression of DLBCL patients in the high- and low-risk groups classified by the 9-ELncRNA signature in the TMUCIH validation cohort. (B) A nomogram was constructed using high and low ELncSig scores, age, LDH, GCB vs. non-GCB, ECOG and extranodal sites to predict 1-, 3- and 5-year survival. (C) Calibration plots for the probability of five-year survival in the training cohort. (D) Time-dependent ROC curve analyses for predicting OS at 5 years with clinicopathological characteristics. [file Image_4.tif]

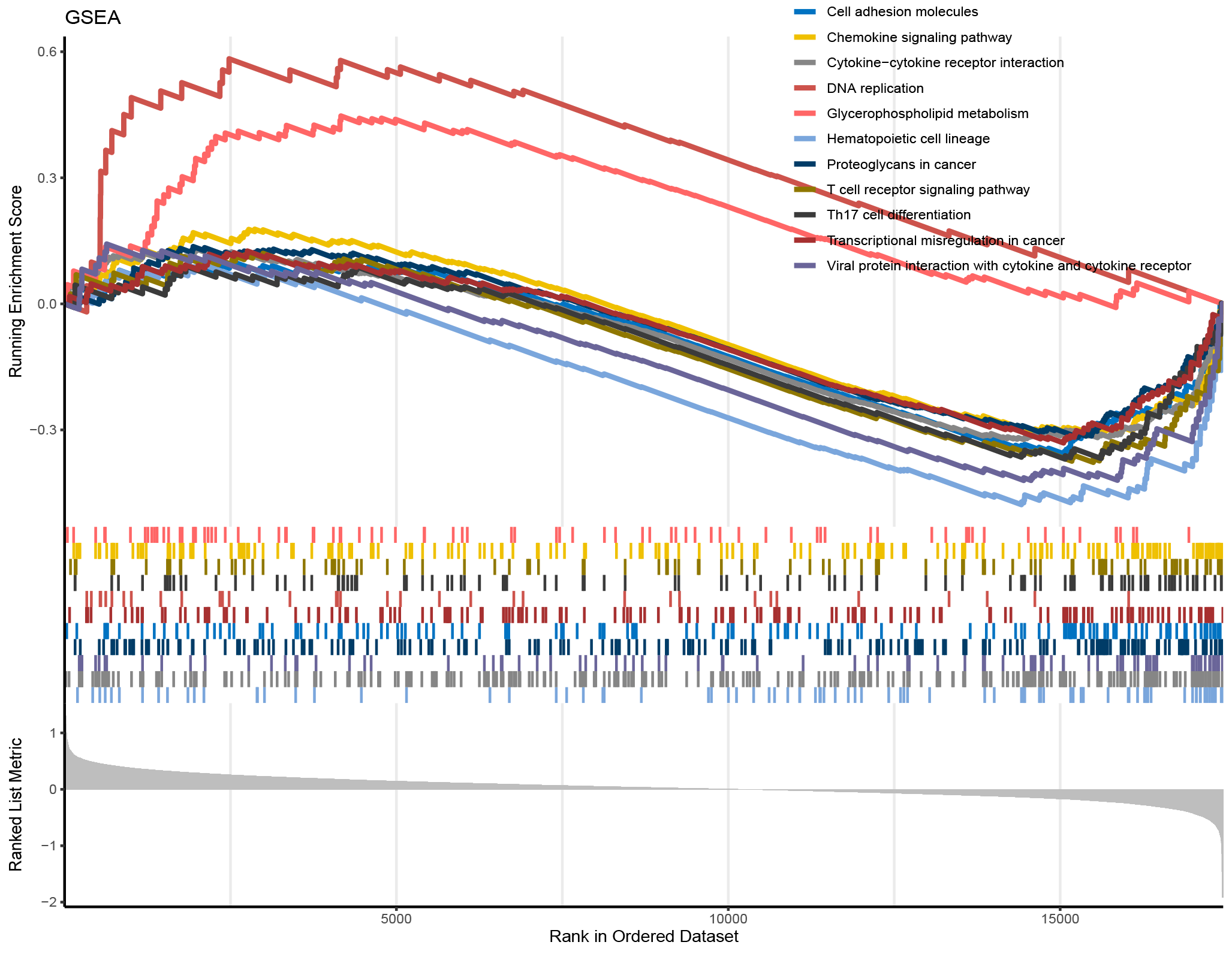

Supplement: Supplementary Figure 5 — GSEA of the DEGs in the training cohort. [file Image_5.tif]

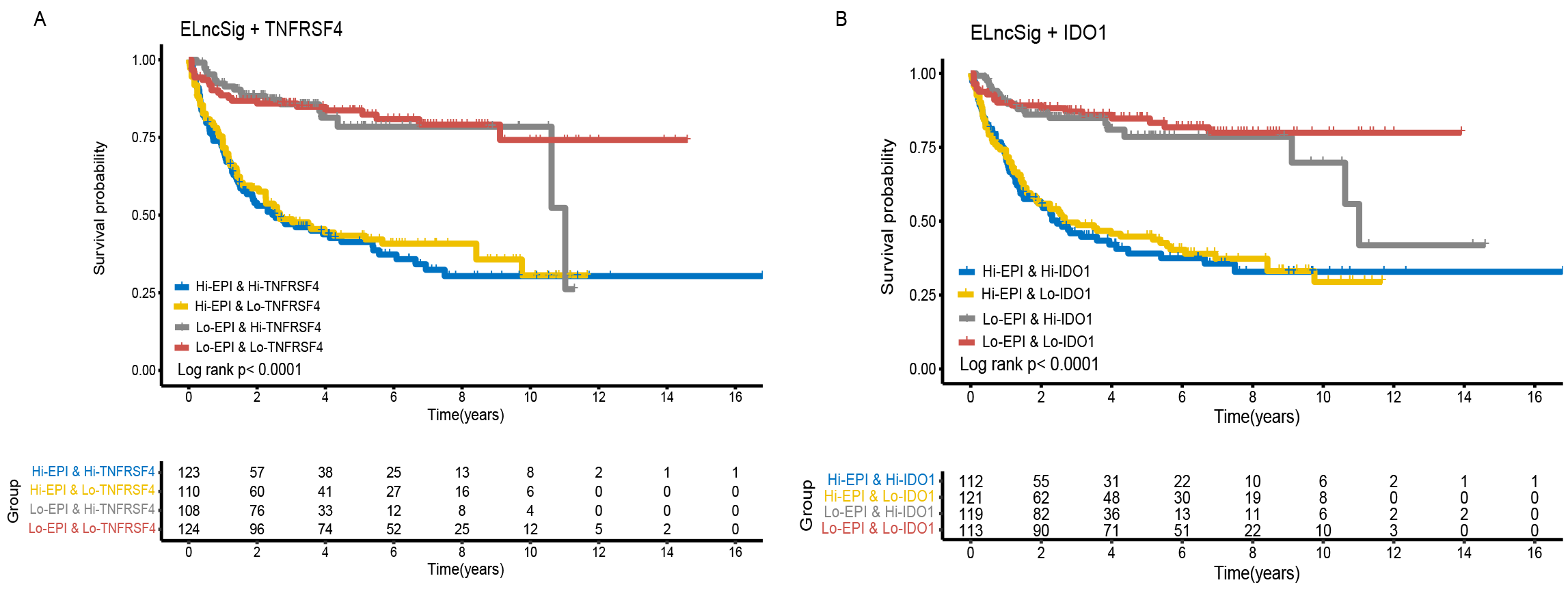

Supplement: Supplementary Figure 6 — Immune checkpoint genes related to clinical outcome. (A, B) Kaplan–Meier survival curves of overall survival among four patient groups stratified by ELncSig and TNFRSF4 and IDO1. [file Image_6.tif]

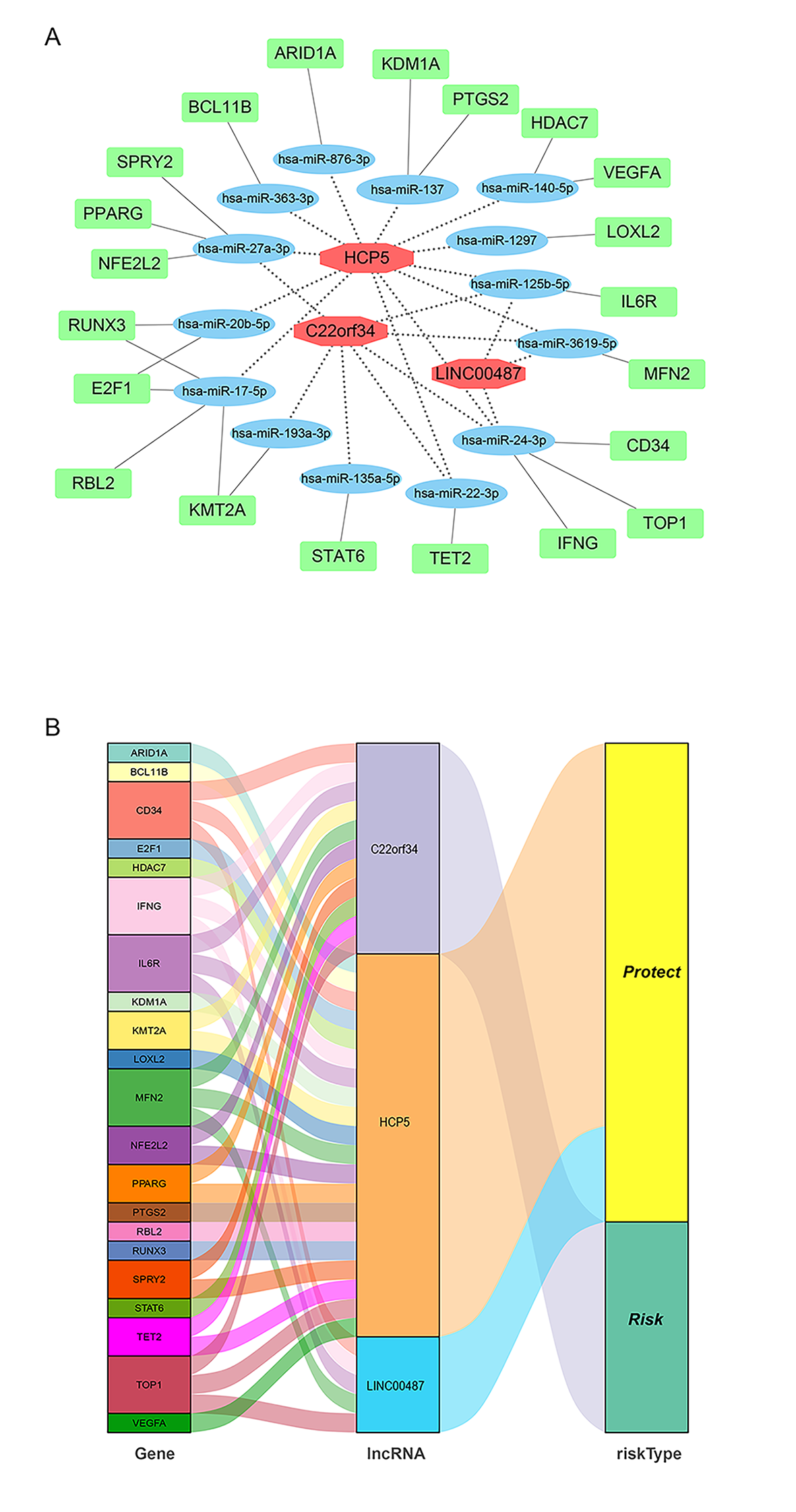

Supplement: Supplementary Figure 7 — Coexpression network and validation of prognostic ELncSig lncRNAs and the associated epigenetic-related genes. (A) A coexpression network of ELncSig lncRNAs and mRNAs (only epigenetic-related genes) was constructed and visualized using Cytoscape. Red hexagons indicate prognostic lncRNAs, and green rectangles indicate ELncSig mRNAs. (B) Sankey diagram showing the associations among prognostic ELncSig lncRNAs, mRNAs (only epigenetic-related genes), and risk type. [file Image_7.tif]
